# Microalgae as Sustainable Renewable Energy Feedstock for Biofuel Production

**DOI:** 10.1155/2015/519513

**Published:** 2015-03-22

**Authors:** Srikanth Reddy Medipally, Fatimah Md. Yusoff, Sanjoy Banerjee, M. Shariff

**Affiliations:** ^1^Laboratory of Marine Biotechnology, Institute of Bioscience, Universiti Putra Malaysia (UPM), 43400 Serdang, Selangor, Malaysia; ^2^Department of Aquaculture, Faculty of Agriculture, Universiti Putra Malaysia (UPM), 43400 Serdang, Selangor, Malaysia; ^3^Department of Veterinary Clinical Studies, Faculty of Veterinary Medicine, Universiti Putra Malaysia (UPM), 43400 Serdang, Selangor, Malaysia

## Abstract

The world energy crisis and increased greenhouse gas emissions have driven the search for alternative and environmentally friendly renewable energy sources. According to life cycle analysis, microalgae biofuel is identified as one of the major renewable energy sources for sustainable development, with potential to replace the fossil-based fuels. Microalgae biofuel was devoid of the major drawbacks associated with oil crops and lignocelluloses-based biofuels. Algae-based biofuels are technically and economically viable and cost competitive, require no additional lands, require minimal water use, and mitigate atmospheric CO_2_. However, commercial production of microalgae biodiesel is still not feasible due to the low biomass concentration and costly downstream processes. The viability of microalgae biodiesel production can be achieved by designing advanced photobioreactors, developing low cost technologies for biomass harvesting, drying, and oil extraction. Commercial production can also be accomplished by improving the genetic engineering strategies to control environmental stress conditions and by engineering metabolic pathways for high lipid production. In addition, new emerging technologies such as algal-bacterial interactions for enhancement of microalgae growth and lipid production are also explored. This review focuses mainly on the problems encountered in the commercial production of microalgae biofuels and the possible techniques to overcome these difficulties.

## 1. Introduction

World energy crisis and global warming are the two major problems human kind faces today, which are mainly due to the more population growth, fast industrialization, and increased use of fossil fuels [1]. Hence, the importance for identification of potential renewable source for sustainable energy production has gained momentum recently [2]. Currently many countries are using biomass, waste, solar, wind, hydro and geothermal energy sources as alternative to fossil based fuels [3]. International Energy Agency (IEA) recently declared that the energy from wastes and combustible sources has higher potential as alternative fuel as compared to other renewable sources [4].

Biofuel (biodiesel, bioethanol, and biogas) from combustible sources is presently being recognised as an alternate and green renewable fuel for sustainable energy production in the near future [5]. Microalgae are the photosynthetic microorganisms, which are attracting huge interest from researchers, government, and local and international entrepreneurs. Recently, the usage of liquid biofuels such as biodiesel, bioethanol, and jet fuel has increased immensely especially in the transport industry [6]. Compared to fossil diesel, biodiesel has many advantages such as it is biodegradable and nontoxic and it has lower emissions of greenhouse gases (GHG) [7].

Microalgae biofuels belong to the third generation type of biofuels, which are considered as an alternative energy source for fossil fuels without the disadvantages associated with the first and the second generation biofuels [8]. Generally the first generation biofuels are derived from crop plants, such as soybean, corn, maize, sugar beet, and sugar cane; palm oil; rapeseed oil; vegetable oils; and animal fats [8]. These types of biofuels have created a lot of disputes due to their negative impacts on food security, global food markets, water scarcity, and deforestation [9, 10]. In addition, the second generation biofuels derived from nonedible oils (*Jatropha curcas*,* Pongamia pinnata, Simarouba glauca, *etc.), lignocellulose biomass, and forest residues require huge areas of land otherwise that could be used for food production. Currently, the second generation biofuel production also lacks efficient technologies for commercial exploitation of wastes as source for biofuel generation [6]. Based on the above-mentioned drawbacks associated with the first and second generation biofuels, microalgae biofuel seems to be a viable alternative source of energy to replace or supplement the fossil fuels.

Several species of microalgae, such as* Botryococcus braunii*,* Nannochloropsis* sp.,* Dunaliella primolecta*,* Chlorella* sp., and* Crypthecodinium cohnii, *produce large quantities of hydrocarbons and lipids.* Botryococcus braunii,* the colonial green microalgae, has the capability to produce a large number of hydrocarbons as compared to its biomass, and it also synthesizes other commercially important compounds such as carotenoids and polysaccharides [11–16]. The production level of oil content in microalgae species reaches up to 80% and the levels from 20 to 50% are quite common [16–18]. The microalga* Chlorella* has up to 50% lipids and* B. braunii* produces the highest oil content of approximately 80% [17]. In Table 1, a comparison was given between the oil yield, production, and biodiesel productivity of microalgae with some other biofuel feedstock. Other than biofuel, microalgae also synthesize different bioactive compounds and have varied applications in nutraceuticals, pharmaceuticals, and chemical and food industries [19, 20].

Microalgae biofuel production is commercially viable because it is cost competitive with fossil based fuels, does not require extra lands, improves the air quality by absorbing atmospheric CO_2_, and utilizes minimal water [23]. However, microalgae biofuels have some disadvantages such as low biomass production, low lipid content in the cells, and small size of the cells that makes harvesting process very costly. These limitations can be overcome by improving the technologies for harvesting and drying and genetic engineering of metabolic pathways for high growth rate and increased lipid content. Initial evaluation of microalgae as the potential source for biofuel production began in 1970, but it was temporarily shelved due to technical and economic problems [24]. Later, subsequent studies from 1980 onwards showed high potential in microalgae biofuel production [25].

## 2. World Market for Biofuel Production 

Large scale commercial production of microalgae began in Japan in the early 1960s by culturing* Chlorella* as food additive. Later, in the 1970s and 1980s it expanded to reach other countries such as USA, Australia, India, and Israel. By the year 2004, the microalgae industry had grown to produce 7000 tonnes of dry matter per annum [26–28].

Biofuel production in the world has increased recently, mainly in the production of bioethanol from sugar crops (e.g., sugar cane, sugar beet, and sweet sorghum) and cereals (wheat and maize). World bioethanol production in 2009 was 73.9 billion liters which showed 400% increase as compared to that in 2000, which was only 17 billion liters [29]. Based on this progress, the global bioethanol production in 2017 will be double that of 2007 [30]. United States and Brazil remained the top most bioethanol producers in the world. In Brazil, with the exception of bioethanol from sugar cane, other biofuels are economically not competitive with fossil based biofuels without subsidies [10].

The world biodiesel production in the year 2003 was around 1.8 billion litres [31]. Countries like United States, Brazil, Canada, China, India, and Japan and Europe are motivated to develop internal biofuel markets and the plans were established to use these biofuels. During the past several years, an increase in biodiesel production was observed because of the increased demand for fuels, to produce “cleaner” energy globally, to fulfil the Bali Action Plan and Kyoto Protocol requirements and establishment of alternative sources for agricultural producers [32]. Currently, the biodiesel production rates in Southeast Asian countries such as in Malaysia, Thailand, and Indonesia range between 70 and 250% [33].

Europe is also an important biofuel producer in the global market. Currently, the European Union countries have a small share (6%) in global biofuel production. The European production levels in global market have increased 40 times from 80 tons in 1993 to 780 tons in 2001 and 3.184.000 tons in 2005 [34]. Germany is the top most biofuel producing country in Europe followed by France, Italy, and Czech Republic [35]. In Europe, biodiesel production occupies the top position (79.5%) among liquid biofuels in the year 2004 [34]. In European countries, biodiesel is generally used by applying various blends with diesel. However in some countries like Germany, Sweden, and Austria, pure biodiesel is used in adapted captive fleet vehicles. Presently in Europe about 1.4 million hectares of arable land is dedicated for biodiesel production. At the moment there are about 40 plants in the EU producing up to 3,184.00 tonnes of biodiesel yearly; and these plants are located mainly in Italy, Germany, Austria, Sweden, and France.

## 3. Production of Microalgae Biomass and Biofuel 

**Figure 1:**
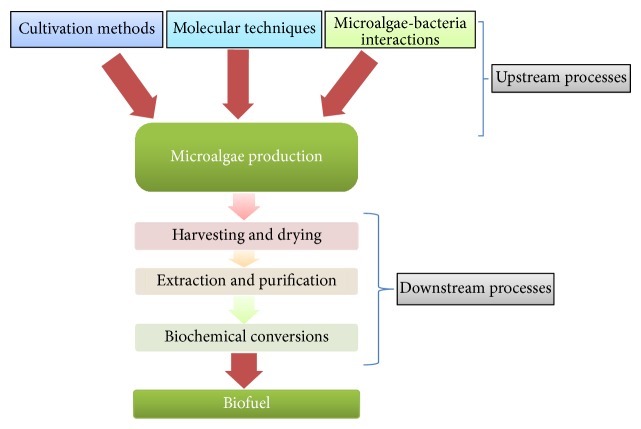
Different strategies involved in microalgae biomass and biofuel production.

Microalgae biomass and biofuel production can be developed at two major phases that involve upstream and downstream processes (Figure 1). The upstream phase involves different cultivation technologies to maximize biomass quality and quantity, whereas the downstream stage puts emphasis on harvesting technologies and sustainable production of biofuel.

### 3.1. Upstream Processes

#### 3.1.1. Microalgae Cultivation Technologies

Production of microalgae biomass can be carried out by three different types of culture systems such as batch, semi-batch, and continuous systems. The growth rate and maximum biomass production of microalgae strains in these culture systems are affected by abiotic (light, temperature, pH, salinity, O_2_, CO_2_, nutrient stress, and toxic chemicals), biotic (pathogens and competition by other algae), and operational (shear produced by mixing, dilution rate, depth, harvest frequency, and addition of bicarbonate) factors. A number of studies have been conducted to develop different cultivation technologies for bulk production of microalgae biomass [28, 36]. Usually, microalgae can be cultivated using four types of cultivation methods such as phototrophic, heterotrophic, mixotrophic, and photoheterotrophic [37] cultivation methods (Table 2). Among these, only phototrophic cultivation is commercially feasible for large scale microalgae biomass production [26]. In addition to this, phototrophic microalgae can also capture atmospheric carbon dioxide and act as a potential carbon sink.

*(1) Phototrophic Cultivation*. Microalgae have high photosynthetic efficiency and growth rates when compared to higher plants [16]. In phototrophic method, microalgae can generally be cultivated in open ponds and enclosed photobioreactors.

*(a) Open Pond Production*. These are the oldest and simplest systems commonly used for large scale microalgae production. These microalgae cultivating methods have been practiced since the 1950s [26]. Presently, about 98% of commercial algae are produced in these systems [41]. There are various types of open pond systems which are mainly differentiated based on their size, shape, and material used for construction, type of agitation, and inclination [42]. Some common ones include raceways stirred by a paddle wheel, extensive shallow unmixed ponds, circular ponds mixed with a rotating arm, and sloping thin-layer cascade systems. Among the above-mentioned systems, raceways are the most commonly used artificial system [43]. Open pond system is the cheapest method for large scale cultivation of microalgae compared to close PBRs. Open pond systems do not compete with agricultural crops for land, since they can be established in minimal crop production areas [44]. The construction, regular maintenance, and cleaning of these systems are easy and they also consume relatively low energy [45]. Open pond systems are less technical in design and are more scalable; however, they are limited by abiotic growth factors like temperature, pH, light intensity, and dissolved oxygen concentration and are easily subjected to contamination [46]. Contamination from the air and ground is often a serious limiting factor for cultivation of algae in open pond systems and therefore most of the species cultured in that systems are grown under selective environments such as high alkalinity and high salinity [47–50].

*(b) Enclosed Photobioreactors (PBR)*. These systems are generally available in the form of tubes, bags, or plates, which are made up of glass, plastic, or other transparent materials. Algae are cultivated in these systems with adequate supply of light, nutrients, and carbon dioxide [51, 52]. Although many PBR designs are available, only a few are practically used for bulk production of algae [53]. Some common PBR designs include annular, tubular, and flat-panel reactors, with large surface areas [52, 54].

*Annular Photobioreactors*. These photobioreactors are more frequently used as bubble columns or airlift reactors [55]. But occasionally they are used as stirred tank reactors [56]. Generally in column photobioreactors the columns are arranged vertically, and aeration is provided from below, and light illumination is supplied through transparent walls. Column photobioreactors have the advantages of best controlled growth conditions, efficient mixing, and highest volumetric gas transfer rates [57].

*Tubular Photobioreactors*. In these reactor systems the algal cultures are pumped through long and transparent tubes. The mechanical pumps or airlifts create the pumping force, and the airlift also allows the exchange of CO_2_ and O_2_ between the liquid medium and the aeration gas [58–61].

*Flat-Panel Photobioreactor*. Flat-panel photobioreactors support higher growth densities and promote higher photosynthetic efficiency [45, 62]. In flat-panel system, a thin layer of more dense culture is mixed or sailed across a flat clear panel; and the incoming light is absorbed within the first few millimetres at the top of the culture [63–65].

As compared to open pond systems, photobioreactors have many advantages such as controllable growth, system efficiency, and algal purity. However, there are some disadvantages such as high costs of construction, operation, and maintenance (Table 3). Though these drawbacks can be partially compensated by higher productivities, they still limit the cost-effective production of microalgae biomass on required scale for biodiesel production.

*(c) Hybrid Production Systems*. In these hybrid systems both open ponds and close photobioreactors are used together in combination to get better results. In these systems, the required amount of contamination free inocula obtained from photobioreactors is transferred to open ponds or raceways to get maximum biomass yield [72, 73]. Olaizola [74] and Huntley and Redalje [75] used these hybrid systems for the production of astaxanthin from* Haematococcus pluvialis*. However this is not suitable for biofuel production because this system is more expensive and it is also a batch culture system rather than a continuous culture system.

*(2) Heterotrophic Cultivation*. In heterotrophic cultivation, instead of photosynthetic process, microalgae utilize organic carbon for their growth and development. As photosynthetic organisms, microalgae are usually light-limited at high cell densities during large scale cultivation [76] or they experience photoinhibition if the light is too intense, both of which lead to slow growth and production [77]. Based on these drawbacks associated with phototrophic cultivation, heterotrophic cultivation of microalgae can be considered favourably [78]. The major advantages associated with heterotrophic cultivation over phototrophic cultivation are the good control on cultivation procedure, elimination of light necessity, and low cost of biomass harvesting [79]. However, heterotrophic cultivation also has some limitations. (1) Limited number of heterotrophic capable species is a limitation. Until now only four types of heterotrophically grown microalgae such as* C. protothecoides* [80–82],* C. vulgaris* [38],* Crypthecodinium cohnii* [83], and* Schizochytrium limacinum* [84] have been identified with high lipid production. (2) Contamination from other organisms is another problem due to the presence of organic substrate [78]. (3) Glucose is the preferred organic substrate for heterotrophic growth of microalgae. However the utilization of plant-based glucose leads to food versus fuel feud because this is also used for human consumption [85]. Therefore, there is a necessity to develop an alternative technology to use lignocellulose and glycerol derived glucose. (4) Generally microalgae release the CO_2_ through respiration but in heterotrophic cultivation it cannot sequester the CO_2_ from atmosphere [86]. Therefore, more comprehensive LCA studies and proactive research for heterotrophic cultivation of microalgae are highly required.

*(3) Mixotrophic Cultivation*. Most of the microalgae utilize both the autotrophic and heterotrophic pathways for their growth and development, indicating that they are able to photosynthesize and utilize organic material [87, 88]. In mixotrophic growth system microalgae cannot depend entirely on photosynthesis because light is not a complete limiting factor, as either light or organic substrate can be utilized for growth [78, 89]. Microalgae which exhibit mixotrophic metabolism are* Spirulina platensis* (cyanobacteria) and* Chlamydomonas reinhardtii* (green algae) [78]. In these organisms, photosynthesis takes place by utilizing light, whereas aerobic respiration uses an organic carbon source for growth [87]. Here the growth of the organism is influenced by the media supplemented with glucose during the light and dark phases; hence, biomass loss during the dark phase is less [89]. A subtype of mixotrophy is called amphitrophy. This type of organisms can survive either autotrophically or heterotrophically, depending on the availability of organic carbon source and light intensity [90].

Chojnacka and Noworyta [91] compared the growth of* Spirulina *sp. in photoautotrophic, heterotrophic, and mixotrophic cultures. Their observation indicated that cultures grown in mixotrophic conditions showed reduced photoinhibition and enhanced growth rates as compared to autotrophic and heterotrophic culture conditions. Therefore, fruitful mixotrophic production of microalgae permits the incorporation of photosynthetic and heterotrophic compounds during diurnal cycle. Mixotrophic cultivation reduces the impact of biomass loss during dark respiration and decreases the utilization of a number of organic matters during growth. Based on these features, mixotrophic cultivation plays a significant role in microalgae biofuel production.

Photoheterotrophy is also known as photometabolism or photoorganotrophy or photoassimilation. In this cultivation system, organic substrate is utilized as carbon source in the presence of light. There is no clear differentiation between photoheterotrophic and mixotrophic metabolisms, but they can specifically be defined according to the requirement of energy source for growth and particular metabolite production [90].

#### 3.1.2. Molecular Strategies to Improve Microalgae Biomass and Biofuel Production

Manipulation of metabolic pathways by using genetic engineering in microalgae is relatively easy due to its unicellular formation. The main objective of applying genetic engineering to microalgae is to improve the biomass and biodiesel production. The progress in genetic engineering of microalgae was extremely slow until recently. Availability of the microalgae genome sequences greatly facilitates the genetic engineering technology. To date genome sequencing projects were completed for several microalgae species [92] and the sequencing projects for some other microalgae species such as* Fragilariopsis cylindrus*,* Pseudo-nitzschia*,* Thalassiosira rotula*,* Botryococcus braunii*,* Chlorella vulgaris*,* Dunaliella salina*,* Galdieria sulphuraria* and* Porphyra purpurea* are under progress [93, 94]. In addition to this, several sequencing projects for different species of microalgae plastids and mitochondria were completed and some projects are continuing [92, 95–97]. The development of methodologies for microalgae genetic transformation has progressed considerably in the last 15 years. Advanced methodologies were developed for green, red, and brown algae, diatoms, euglenoids, and dinoflagellates, and until now 30 microalgae strains have been successfully transformed [92]. Most of the transformation experiments were made on a model green alga* Chlamydomonas reinhardtii* at both nuclear and chloroplast levels [98, 99].

*(1) Genetic Engineering for Enhanced Biomass Production*. Microalgae growth is generally influenced by various environmental stress conditions such as temperature, light, salt concentration, and pH. These conditions can be controlled by engineering and manipulations of growth characteristics, but these manipulations increase the total growing costs of microalgae. Thus, it will be beneficial if the genetic engineering strategies can be developed to control these environmental stress conditions. The average light intensity which provides the maximum photosynthesis in most microalgae species is around 200–400 *μ*M photons m^−2^s^−1^. The light intensity above this level reduces the microalgae growth. During midday, the maximum light intensity reaches up to 2,000 *μ*M photons m^−2^s^−1^ [100]. Because of this, microalgae growth efficiency during day time is less. Therefore, several studies were carried out to improve the microalgae photosynthetic efficiency and also to reduce the effect of photoinhibition. Most of these studies were carried out by reducing the number of light-harvesting complexes (LHC) or lowering the chlorophyll antenna size to decrease light absorbing capacity of individual chloroplasts [101]. In an experiment, LHC expression in transgenic* C. reinhardtii* was downregulated to increase the resistance to photooxidative damage and to enhance the efficiency of photosynthesis by 50% [101, 102]. This alteration allowed* C. reinhardtii* to tolerate photoinhibition. In another study conducted by Huesemann et al. [103], no growth improvement was observed in algal antenna mutants cultured in outdoor ponds and also in laboratory conditions. Genes that are able to withstand other stress conditions such as temperature, pH, salt concentration, and other stimuli have also been identified.

*(2) Genetic Engineering for Enhanced Biofuel Production*. Genetic engineering application in the improvement of microalgae biofuel production is still in the initial stage. Some important advances have been made in the past few years such as development of genetic transformation strategies; sequencing of nuclear, mitochondrial, and chloroplast genomes; and establishment of expressed sequence tag (EST) databases [92]. The current molecular strategies required to improve microalgae biodiesel production include blocking energy rich compounds (e.g., starch) producing metabolic pathways, to decline lipid catabolism, that is, elimination of fatty acid *β*-oxidation that consumes TAGs; modification of lipid characteristics; direct biological synthesis of fatty acids; and secretion of TAGs, free fatty acids, alkane, and wax esters directly into the medium [92].

#### 3.1.3. Interactions with Bacterial Biofilms to Improve Biomass and Biofuel Production

**Figure 2:**
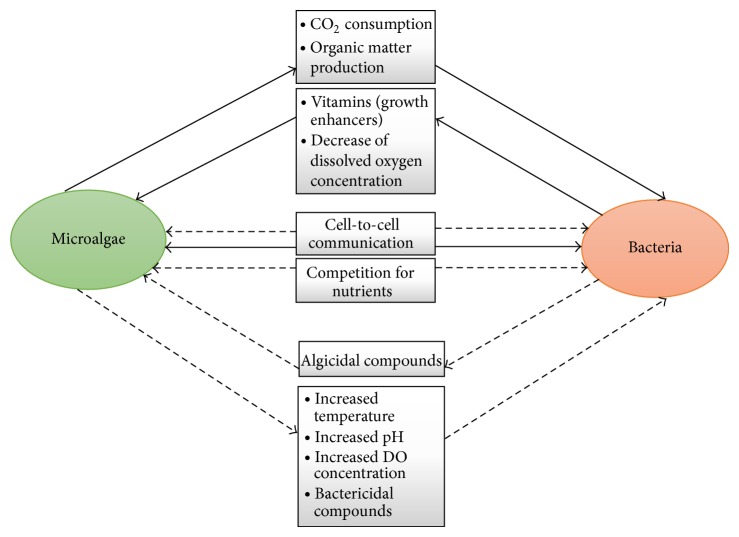
Possible interactions between microalgae and bacteria: solid arrows indicate the positive interactions and dashed arrows indicate the negative interactions [118, 119].

Microalgae and bacteria perform symbiotic relationship by establishing “phycosphere” [104, 105] as plants and bacteria do in the “rhizosphere” [106]. Microalgae produce extracellular products for the development of matrix like substance on their surfaces, which encourages and provides the environment for the formation of bacterial biofilms [107, 108]. Teplitski et al. [109] reported the existence of microalgae-bacteria interactions in the unicellular microalgae* Chlamydomonas reinhardtii*. To date, only limited studies have been carried out about the existence of interactions between bacterial biofilms and microalgae [110–112]. These studies suggest that the bacteria encourage the growth of microalgae by producing the vitamins and other growth factors, and the organic matters produced by the microalgae simultaneously encourage bacterial growth. They also have negative interactions between each other; microalgae inhibit the bacterial growth by increasing the temperature, pH, and dissolved oxygen concentration (DOC) or by producing inhibitory metabolites [113, 114], and in the same manner bacteria also can inhibit the microalgae growth by secreting algicidal compounds [115] (Figure 2). Recent reports suggested that the presence of these positive interactions between microalgae and bacteria enhances the microalgae biomass and biodiesel production [116, 117].

### 3.2. Downstream Processes

#### 3.2.1. Harvesting and Drying of Microalgae Biomass

After attaining sufficient biomass, the microalgae cells are separated from water and prepared for downstream processing. Generally one or more solid-liquid separation steps are required for microalgae biomass separation [23, 120, 121]. According to life cycle analysis, this separation process accounts for 20–30% of the total biofuel production costs [122]. Biomass harvesting and drying processes may constitute major energy consumption in microalgae biofuel production [123]. Therefore, there is a need to reduce energy consumption in microalgae biomass harvesting and drying processes; otherwise, it may cause major cost increase in the overall processes of microalgae biofuel production [124, 125].

#### 3.2.2. Extraction and Purification of Lipids from Microalgae Biomass

Several methods such as presses, supercritical carbon dioxide extraction, ultrasonic-assisted extraction, osmotic shock, solvent extraction, and enzymatic extraction are available for oil extraction from microalgae biomass. The first three methods are used only at laboratory scale. The most important aspects to be considered for selection of appropriate oil extraction process are the cost, efficiency, toxicity, and ease of handling. Supercritical carbon dioxide and osmotic shock are not commercially viable methods due to high operation costs [126]. Enzymatic extraction method is commercially possible, but some efforts are needed to reduce the costs [127, 128]. However, some commercially viable methods are needed to minimize the cost, maximize the extraction of desirable lipid fractions, and reduce the coextraction contaminants.

#### 3.2.3. Microalgae Biomass Conversion Technologies

**Figure 3:**
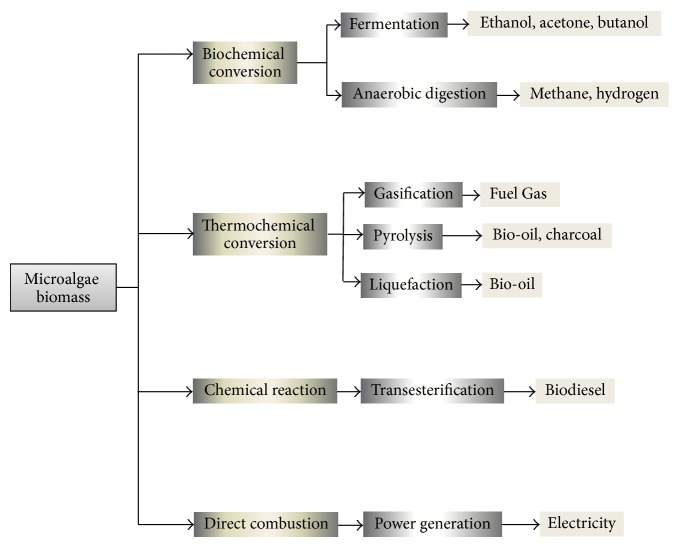
Microalgae biomass conversion processes [23, 135].

Microalgae biomass conversion technologies are classified into different types such as biochemical conversion, thermochemical conversion, chemical reaction, and direct combustion [129] (Figure 3). Biochemical conversion can be applied to produce methanol (anaerobic digestion) and ethanol (fermentation) from microalgae biomass [28]. Thermochemical conversion processes can be categorised into pyrolysis (bio-oil, charcoal), gasification (fuel gas), and liquefaction (bio-oil) [130–132]. The energy stored in microalgae cells can be converted into electricity by using direct combustion process. In chemical conversion technologies transesterification process can be employed for the conversion of extracted lipids into biodiesel [16]. Transesterification process is quite a sensitive process as it depends on different parameters such as free fatty acids (FFAs), water content, molar ratio of alcohol to oil, catalyst, reaction temperature, and stirring [133]. Catalytic processes are more appropriate in converting biomass to biodiesel, especially nanocatalysts which have the good capacity in improving product quality and attaining best operating conditions [134].

## 4. Limitations of Biofuel Production from Microalgae

In addition to many advantages, microalgae biofuels also have some disadvantages. The main limitations involved in microalgae biofuel production are the low concentration of biomass in the culture and low oil content. In addition, small size of microalgae cells makes the harvesting process quite costly. Harvesting and drying of microalgae biomass from high volume of water are an energy consuming process. Compared to the conventional agriculture practice, microalgae farming is more costly and complicated. These difficulties can be minimized or overcome by the improvement of the harvesting technology. Some of the cost effective technological strategies suggested to develop microalgae biofuel production are (1) development of biorefinery or coproduct strategy, (2) designing high photosynthesis efficiency photobioreactors, (3) development of cost-effective technologies for biomass harvesting and drying, (4) development of genetic engineering technology to modify metabolic pathways for microalgae biomass and lipid production, and (5) understanding of symbiotic interactions between microalgae and bacteria that also affects the biomass and lipid production in microalgae.

## 5. Economic Analysis of Microalgae Biofuel Production

Economy plays an important role in the commercial feasibility of microalgae biofuel production [136]. Microalgae oil production cost depends on various factors, such as biomass yield, oil content, scale of production systems, and cost of recovering oil from microalgae biomass. It also depends on the petroleum oil price. According to Gallagher [137], the economic feasibility of microalgae biofuel production seems to be fair and dependent on government subsidies and the future prices of oil. In addition to optimized biomass yields, the requirement of carbon neutral renewable alternatives makes microalgae one of the best future sources of biofuels [16]. Norsker et al. [138] calculated biomass production costs for three different commercial production systems such as open ponds, horizontal tubular photobioreactors, and flat-panel photobioreactors. The resulting biomass production costs for these three systems including dewatering were 4.95, 4.15, and 5.96 € per kg, respectively. The factors which influence production costs are irradiation, mixing, photosynthetic efficiency, culture medium, and CO_2_. If we optimize these factors, the production cost reduces to € 0.68 per kg and at this cost microalgae become promising feedstock for biodiesel production and for other applications. Generally the following formula can be used to estimate the cost of algal oil where it can be a competitive substitute for petroleum diesel [16]:

(1) Calgal  oil=25.9×10−3Cpetroleum,

where *C*_algal  oil_ is the microalgae oil price in dollars per gallon and *C*_petroleum_ is the crude oil price in dollars per barrel.

According to the above-mentioned formula, the algal oil roughly contains 80% of the caloric energy value of crude petroleum. For example, in order to maintain competitiveness with petroleum diesel microalgae oil should not be priced more than $ 0.70/L, if petroleum price is $ 0.62/L.

The biodiesel competitiveness depends mainly on the microalgae biomass production costs. Competitiveness can be calculated by estimating the maximum price that could be paid for microalgae biomass with a given content of oil, if crude petroleum can be purchased at a given price as a source of energy. This estimated price can then be compared with the current cost of producing the algal biomass. According to Chisti [44] the quantity of algal biomass (*M*, tons), which is the energy equivalent to a barrel of crude petroleum, can be estimated as follows:

(2) M=Epetroleumq1−WEbiogas+YWEbiodiesel,

where *E*_petroleum_ (~6100 MJ) is the energy contained in a barrel of crude petroleum, *q* (m^3^ton^−1^) is biogas volume produced by anaerobic digestion of residual algal biomass, *W* is the oil content of the biogas, *Y* is the yield of biodiesel from algal oil, and *E*_biodiesel_ is the average energy content of biodiesel.

Typically, *Y* in (2) is 80% by weight and *E*_biodiesel_ is ~37,800 MJ per ton. Keeping with average values for organic wastes, *E*_biogas_ and *q* are expressed to be around 23.4 MJm^−3^ and 400 m^3^ton^−1^, respectively. Using these values in (2), *M* can be calculated for any selected value of *W*.

Assuming that converting a barrel of crude oil to various useable transport energy products costs roughly the same as converting *M* tons of biomass to bioenergy, the maximum acceptable price that could be paid for the biomass would be the same as the price of a barrel of crude petroleum; thus,

(3) Acceptable  price  of  biomass  $.ton  =Price  of  a  barrel  of  petroleum$M.

By using these equations the prices of microalgae biomass can be estimated for biomass with different levels of oil content (15%–55% by weight). The feasibility of microalgae biofuel can be enhanced by designing advanced photobioreactors, developing cost-effective technologies for biomass harvesting and drying, improving molecular strategies for more biomass and lipid production, and understanding of biotic and abiotic interactions with algae.

## 6. Conclusions

Microalgae have the potential to be important and sustainable renewable energy feedstock that could meet the global demand. In spite of the many advantages, microalgae biofuels also have some disadvantages such as low biomass production and small cell size that makes the harvesting process costly. These limitations could be overcome by designing advanced photobioreactors and developing low cost technologies for biomass harvesting, drying and oil extraction. In addition, application of genetic engineering technology in the manipulation of microalgae metabolic pathways is also an efficient strategy to improve biomass and biofuel production. Genetic engineering technology also plays an important role in the production of valuable products with minimal costs. Biotic interaction with bacterial biofilms is also an important aspect in microalgae biomass and biofuel production. However, these technologies are still in the early stages and most have not been applied on a commercial scale. Therefore, further research in the development of novel upstream and downstream technologies will benefit the commercial production of biofuels from microalgae.

## Figures and Tables

**Table 1:** Comparison of oil content, oil yield, and biodiesel productivity of microalgae with the first and the second generation biodiesel feedstock source [17, 18, 21, 22].

Feedstock source	Oil content (% oil by wt. in biomass)	Oil yield (oil in litres/ha/year)	Biodiesel productivity (kg biodiesel/ha/year)
Oil palm	36	5366	4747
Maize	44	172	152
Physic nut	41–59	741	656
Caster	48	1307	1156
Microalgae with low oil content	30	58,700	51,927
Microalgae with medium oil content	50	97,800	86,515
Microalgae with high oil content	70	136,900	121,104

**Table 2:** Biomass and lipid productivities of some microalgae under phototrophic, heterotrophic, and mixotrophic conditions.

Cultivation method	Microalgae	Biomass productivity (g L^−1^ d^−1^)	Lipid content (% dry weight biomass)	Lipid productivity (mg L^−1^ d^−1^)	Reference
Phototrophic method	*Chlorella vulgaris *	0.02–0.20	50–58	11.2–40	
	*Chlorella protothecoides *	2.00–7.70	14.6–57.8	1214	[18]
	*Chlorella sorokiniana *	0.23–1.47	19.0–22.0	44.7	

Heterotrophic method	*Chlorella vulgaris *	0.15	23	35	[38]
	*Chlorella protothecoides *	3.1–3.9	—	2400	[39]
	*Chlorella sorokiniana *	1.48	23.3	—	[40]

Mixotrophic method	*Chlorella vulgaris *	0.25–0.26	20.0–22.0	52.0–56.0	
	*Chlorella protothecoides *	23.9	58.4	11,800	[37]
	*Chlorella sorokiniana *	0.58	—	29.0–56.0	

**Table 3:** Comparison between open ponds and photobioreactors [51, 52, 58, 66–71].

Factor	Open ponds	Photobioreactors
Area-to-volume ratio	Large	Small
Algal species	Restricted	Flexible
Species selection	Growth competition	Shear resistance
Sterility	Low	High
Cultivation period	Limited	Extended
Water loss through evaporation	Possible	Prevented
Controlling of growth conditions	Very difficult	Easy
Light utilization efficiency	Poor/fair	Fair/excellent
Gas transfer	Poor	Low-high
Temperature	Highly variable	Required cooling
Temperature control	None	Excellent
Automatic cooling system	None	Built in
Automatic heating system	None	Built in
Cleaning	Not required	Required due to wall growth and dirt
Weather dependence	High	Medium
Process control and reproducibility	Limited	Possible within certain tolerance
Microbiology safety	None	UV
Harvesting efficiency	Low	High
Population density	Low	High
Biomass productivity	Low	High
Biomass quality	Variable	Reproducible
Air pump	Built in	Built-in
Hydrodynamic stress on algae	Difficult	Easy
Shear	Low	High
CO_2_ transfer rate	Poor	Excellent
Mixing efficiency	Poor	Excellent
Volumetric productivity	High	Low
Water loss	Very high	Low
O_2_ concentration	Low due to continuous spontaneous out gassing	Exchange device
CO_2_ loss	High	Low
Land required	High	Low
Capital investment	Small	High
Periodical maintenance	Less	More
Operating cost	Lower	Higher
Harvesting cost	High	Lower
Most costly parameters	Mixing	O_2_, temperature control
Scale-up technology for commercial level	Easy to scale up	Difficult in most PBR models
